# Effect of Estrogen Receptor Status on Circulatory Immune and Metabolomics Profiles of HER2-Positive Breast Cancer Patients Enrolled for Neoadjuvant Targeted Chemotherapy

**DOI:** 10.3390/cancers12020314

**Published:** 2020-01-29

**Authors:** Alessia Vignoli, Elena Muraro, Gianmaria Miolo, Leonardo Tenori, Paola Turano, Emanuela Di Gregorio, Agostino Steffan, Claudio Luchinat, Giuseppe Corona

**Affiliations:** 1 Magnetic Resonance Center (CERM), University of Florence, 50019 Florence, Italy; vignoli@cerm.unifi.it (A.V.); tenori@cerm.unifi.it (L.T.); turano@cerm.unifi.it (P.T.); 2 Consorzio Interuniversitario Risonanze Magnetiche di Metallo Proteine (C.I.R.M.M.P.), 50019 Sesto Fiorentino, Italy; 3 Immunopathology and Cancer Biomarkers Unit, Centro di Riferimento Oncologico di Aviano (CRO), IRCCS, 33081 Aviano, Italy; emuraro@cro.it (E.M.); asteffan@cro.it (A.S.); 4 Medical Oncology and Cancer Prevention Unit, Centro di Riferimento Oncologico di Aviano (CRO), IRCCS, 33081 Aviano, Italy; gmiolo@cro.it; 5 Department of Chemistry, University of Florence, 50019 Florence, Italy

**Keywords:** metabolomics, cytokines, breast cancer, estrogen receptors, HER2-positive

## Abstract

HER2-positive breast cancer (BC) represents a heterogeneous cancer disease. In an attempt to identify new stratification models useful for prognosis and therapeutic strategy, we investigated the influence of estrogen receptor (ER) status on the host immune and metabolomics profile of HER2-positive BC patients enrolled for neoadjuvant targeted chemotherapy (NATC). The study enrolled 43 HER2-positive BC patients eligible for NATC based on the trastuzumab-paclitaxel combination. Baseline circulatory cytokines and ^1^H NMR plasma metabolomics profiles were investigated. Differences in the immune cytokines and metabolomics profile as a function of the ER status, and their association with clinical outcomes were studied by multivariate and univariate analysis. Baseline metabolomics profiles were found to discriminate HER2-positive ER(+) from ER(−) BC patients. Within the ER(+) group an immune-metabolomics model, based on TNF-α and valine, predicted pathological complete response to NATC with 90.9% accuracy (AUROC = 0.92, *p* = 0.004). Moreover, metabolomics information integrated with IL-2 and IL-10 cytokine levels were prognostic of relapse with an accuracy of 95.5%. The results indicate that in HER2-positive BC patients the ER status influences the host circulatory immune-metabolomics profile. The baseline immune-metabolomics assessment in combination with ER status could represent an independent stratification tool able to predict NATC response and disease relapse of HER2-positive patients.

## 1. Introduction

Breast cancer (BC) is one of the most commonly diagnosed cancers worldwide [1]. Over the last decades it has become extremely clear that BC can no longer be considered as a single entity but a highly heterogeneous disease with different subclasses [2,3,4]. The molecular or immunohistochemical investigation enabled the classification of BC into four principal subtypes based on the estrogen receptor (ER), progesterone receptor (PgR), human epidermal growth factor receptor 2 (HER2) and Ki67 expression [5]. The identification of such molecular subtypes at the time of cancer diagnosis represents a mandatory point for determining the cancer prognosis and the therapeutic strategy.

The HER2-positive BC is a highly aggressive subtype that accounts for about 15–20% of all breast cancers [6]. The introduction of trastuzumab, a monoclonal antibody targeted against HER2 receptor, has represented a major milestone in the therapy of HER2 positive BC that profoundly changed the course of this disease [7]. Alongside other pharmacological regimens, it is the gold standard treatment in both adjuvant and neoadjuvant settings. Despite the improvement in overall survival, there is still a significant fraction of HER2-positive BC patients that do not achieve benefit from the pharmacological treatment and relapse within 5 years from the treatment [6,8]. In the era of precision medicine, such individual failures are calling for the investigation of new patient stratification models able to better predict the clinical outcomes. The HER2-positive, as other BC, constitutes a heterogeneous group which can be subdivided, according to ER status, in HER2-positive/ER(−) and HER2-positive/ER(+) reflecting the stage of differentiation and the cell origin [5]. Overall, ER(−) BC has been known to be significantly different from ER(+) BC in the genetic pattern, type and gene expression complexity [9]. Besides gene expression, metabolomics studies on BC tissues showed also that ER(+) and ER(−) subtypes present distinct cellular metabolism, especially for what concerns the metabolic pathways that involve glutamate/glutamine and beta-alanine [10,11,12].

Metabolomics is a relatively new -omics approach useful to study the complex and dynamic biochemical interplay occurring between cancer and host [13]. Thus, metabolomics not only can potentially identify the biochemical signals directly caused by the presence of cancer, but it can also detect the interaction between cancer and host, which is mainly mediated by the immune system. In the BC field, the potential of metabolomics in blood serum or plasma has been explored to identify new biomarkers useful for early diagnosis and prognosis [14,15,16,17,18,19,20,21,22,23,24].

There are clear evidences on the role of host immune response in mediating the clinical efficacy of NATC especially when used in combination with drugs that have intrinsic immunomodulating properties such as taxanes [25]. In this context, the ER status was found associated with a differential expression of tumor infiltrating lymphocytes (TILs), raising the possibility of different immune-tumor interactions based on ER status [26,27,28]. Moreover, peripheral blood immune cells involved in antibody-dependent cell cytotoxicity (ADCC) and serum cytokines have been also associated to the trastuzumab response [29,30,31,32]. However, the association of response to NATC, as well as of disease relapse, with the ER status appears very elusive, requiring new stratification models to better identify the patients that can receive the best pharmacological benefit. It can be hypothesized that the complexity of the HER2 and ER co-expression and interaction may have a significant impact on the host response, able to influence the pharmacological and clinical outcome as well as the patterns of relapse.

In this study we have investigated for the first time the influence that ER tissue expression may have on the baseline host immune cytokines and nuclear magnetic resonance (^1^H NMR) metabolomic profiles [33,34] in a highly homogeneous population of HER2-positive BC patients enrolled for NATC treatment. We found that ER status might strongly affect the individual circulatory metabolomics profile at baseline, suggesting a differential host–cancer interaction. In the HER2-positive/ER(+) cohort the combination of metabolomics profile with immune serum cytokines resulted useful to identify the good responders (GR), patients who achieved complete pathological response (pCR) to the NATC, as well as to establish the overall risk of disease relapse. These results may pave the way for the integration of the immune-metabolomics profile with conventional histological stratification of HER2-positive BC. 

## 2. Results

### 2.1. HER2-Positive BC Patient Population 

This immune-metabolomics investigation considered 43 HER2-positive BC patients stratified according to their ER status. Thus, a group of 22 patients with ER score higher than 3 constituted the ER(+) patients (luminal B–like subtype) while the remaining 21 with a ER score lower than 3 constituted the ER(−) patients (HER2–enriched subtype). The two groups were highly homogeneous for clinical and pathological characteristics (Table 1). The median age of ER(−) and ER(+) groups was 48 years (range 28–70) and 49 years (range 23–68) respectively. The prevalence of state IIB and grade 3 was high in both groups without significant differences. The rate of pCR to NATC was no significantly different being 13 (48%) and 11 (50%) in ER(−) and ER(+) groups, respectively. At the end of NATC the conserving surgery rate was about 38%. During the follow-up the ER(+) patients received anti-estrogen adjuvant therapy based on tamoxifen or aromatase inhibitors. Within median time follow-up of 10 years the disease relapse occurred in only 3(14%) ER(−) patients and in 8 (36%) ER(+) patients with no statistically significant differences. 

### 2.2. Differential ^1^H NMR Metabolomics Profiles as Function of the ER Status

**Figure 1:**
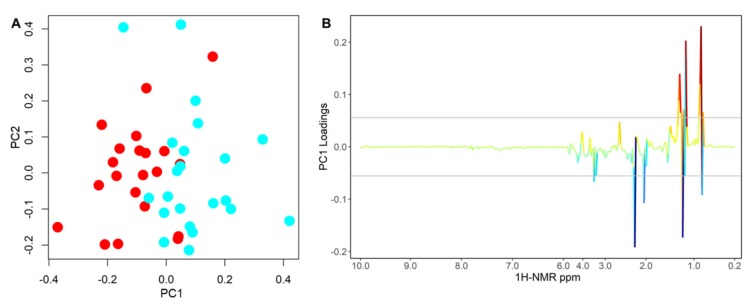
PCA-CA of ^1^H NMR CPMG spectra of baseline plasma samples from HER2-positive ER(+) (cyan) and ER(−) (red) breast cancer patients. Score plot of the first two PCA-CA components (**A**) and loading plot of PC1 (**B**). Model LOOCV accuracy of 74.4%, *p*-value = 0.005.

The differences of baseline plasma metabolomics ^1^H NMR profiles between ER(−) and ER(+) patients were analyzed using PCA-CA-kNN (Figure 1A). To ensure that the calculated model was reliable, and the observed clustering was statistically robust, we performed an internal validation using a Leave-One-Out cross-validation: a good separation of the two groups was obtained yielding 77.3% sensitivity, 71.4% specificity, and 74.4% accuracy. Moreover, comparable results were also obtained with a different Monte Carlo cross-validation. The permutation test for accuracy significance showed a *p*-value of 0.005.

From the loading analysis of the first component of the PCA-CA it emerged that the main sources of discrimination were within the 3.57–3.55 ppm, 3.19–3.17 ppm, 2.23–2.05 ppm, 1.37–1.23 ppm and 0.91–0.83 ppm ^1^H NMR spectral regions. These portions of the spectra are principally related to LDL1 lipoproteins, mainly associated to cholesterol, phospholipids and Apo-B subfractions, and to creatinine signals (Figure 1B). The concentration of these metabolites and lipoproteins were all higher in the ER(+) group as compared to those observed in the ER(−) group (Figure S1).

Significant differences in the metabolomic profile in relation to PgR status and Ki67 were not observed.

### 2.3. Cytokines Immune Profiles as a Function of the ER Status

Serum levels of 10 different cytokines were evaluated at baseline in all the 43 HER2-positive BC patients. Cytokines were selected among the detectable serum factors observed in the peripheral blood of breast cancer patients for their potential role in the modulation of anti-tumor T cell responses. The serum levels of these cytokines for the ER(−) and ER(+) groups are summarized in Figure S2. In particular, ER(+) patients seemed to show higher levels of some T-cell stimulating factors as IL12-p70 and tumor-necrosis factor-α (TNF)-α, compared to ER(−) patients. However, ER(+) patients apparently exhibited also enhanced level of cytokines potentially responsible for the suppression of T-cells as IL-10, IL-8, and transforming growth factor (TGF)-β1, and lower concentrations of both T-cells activating factors (IL-1α and IL-2), and mediators of the inflammatory response as IL-1β, IL-6 and GM-CSF. However, due to the high inter-patient variability and relatively low number of patients enrolled, such differences did not reach statistical significance.

### 2.4. Immuno-Metabolomic Profiles as Predictor of Pharmacological Outcome 

**Figure 2:**
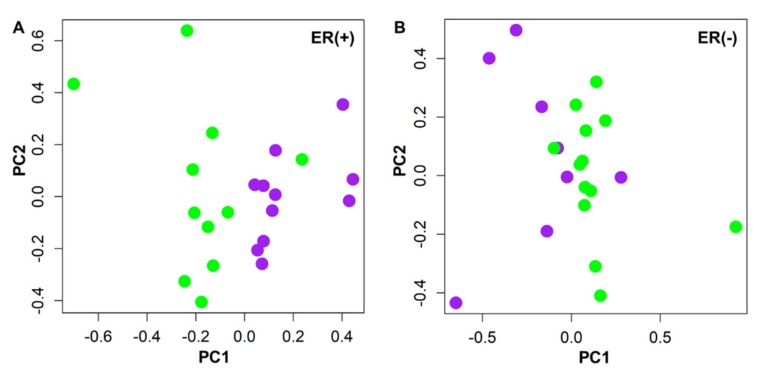
PCA-CA of ^1^H NMR CPMG spectra of baseline plasma samples from patients who achieved complete pathological response (GR, green) and partial responders (PR, purple) to neoadjuvant chemotherapy in ER(+) (**A**) and ER(−) subtypes (**B**). ER(+) model showed LOOCV accuracy of 72.7%, *p*-value = 0.047, ER(−) model showed LOOCV accuracy of 63.2%, *p*-value = 0.143.

Since HER2-positive ER(+) and ER(−) BC groups showed distinct metabolomics profiles, their metabolic responses to NATC were evaluated separately. In the ER(+) group, the ^1^H NMR metabolomics profile was able to discriminate GR patients who achieved pCR from the poor responders (PR) patients by multivariate PCA-CA-kNN analysis, with 63.7% sensitivity, 81.8% specificity, and 72.7% accuracy (Figure 2A and Figure S3A). Conversely, no relevant discrimination between GR and PR patients emerged within the ER(−) group, showing poor classification power: 50.0% sensitivity, 84.6% specificity, and 63.2% accuracy (Figure 2B and Figure S3B). The permutation test for accuracy significance showed a *p*-value of 0.047 and of 0.143 for ER(+) and ER(−) models, respectively.

**Figure 3:**
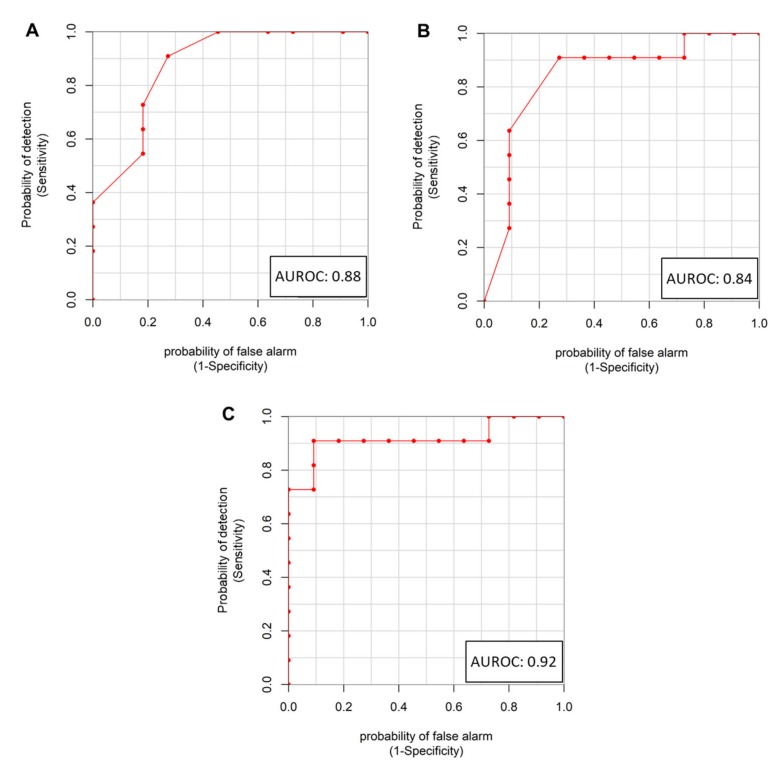
ROC curves of valine (**A**) and TNF-α (**B**) diagnostic power to distinguish ER(+) patients who achieve pathological response (GR) from those who achieve only a partial response (PR) to NATC treatment. The ROC curve of the combined linear model VAL+TNF-α (**C**) shows improved diagnostic power.

The loading analysis of the first component PCA-CA of the ER(+) group revealed that the GR and PR subgroups were mainly discriminated by the 3.99–3.93 ppm, 3.05–3.03 ppm, 2.75–2.71 ppm, 2.05–1.99 ppm, 1.57–1.55 ppm, 1.31–1.23 ppm, 1.05–1.03 ppm and 0.99–0.85 ppm ^1^H NMR spectral regions, corresponding to cholesterol and phospholipid subfractions belonging to ApoB, VLDL, LDL lipoproteins and to isoleucine, valine, and ethanol signals. The GR, as compared to PR patients, are characterized by overall lower concentrations of phospholipids and cholesterol associated to almost all classes of lipoproteins assigned by ^1^H NMR (Figure S4). Analogously, branched-chain amino acids, isoleucine and valine, as well as ethanol, resulted significantly lower in the group of GR as compared to the PR patients. Valine, showing an AUROC = 0.88, a Cliff’s delta effect size of −0.75 (large effect) and a *p*-value of 0.003 (*p* = 0.05 after FDR correction) (Figure 3A) resulted to be the metabolite most significantly associated with the discrimination between GR and PR patients.

The differential cytokines profile analysis between GR and PR within the ER(+) group indicates that TNF-α levels were significantly higher in GR patients, presenting an AUROC = 0.84, a Cliff’s delta effect size of 0.68 (large effect) and a *p*-value of 0.007 (*p* = 0.04 after FDR correction) (Figure 3B, Table S1). Conversely, within the ER(−) subgroup, none of the cytokines and none of the metabolites analyzed was statistically significant in the comparison between GR and PR (Table S2).

A combined immune and metabolomics approach was applied for the ER(+) group in an attempt to obtain a better clustering of GR and PR patients. For this aim we selected two variables, a cytokine (TNF-α) and a metabolite (valine) because, among the measured ones, are those that better discriminated GR and PR to NATC treatment. The linear model combination of the two significant and uncorrelated (R = −0.37, *p*-value = 0.1) variables valine and TNF-α (78.4% TNF-α and 21.6% valine) was found to enhance significantly the GR and PR discrimination, yielding an AUROC = 0.92, a Cliff’s delta effect size of −0.83 (large effect) and a *p*-value of 0.004 (Figure 3C). By setting a threshold that maximized sensitivity and specificity, the valine–TNF-α linear model discriminated the GR from the PR subgroup with an accuracy of 90.9%. The attempts to include in the combined model other less significant variables (i.e., alanine, tyrosine), did not provide any significant improvement. This finding can be interpreted by considering the existence of a correlation with valine. 

Neither valine (AUROC = 0.57, Cliff’s delta effect size of 0.13, *p*-value of 0.92), neither TNF-α (AUROC = 0.51, Cliff’s delta effect size of 0.03, *p*-value of 0.94) resulted significant in the discrimination between GR and PR within the ER(−) group (Figure S5A,B). As a consequence, also their linear combination did not reach the statistical significance (AUROC = 0.59 Figure S5C, Cliff’s delta effect size of 0.18, *p*-value of 0.51). It is worth of mentioning that for 38% of the subjects in the ER(−) group data on TNF-α are missing; surely this lack of information affected the results.

### 2.5. ER(+) Immuno-Metabolomics Profiles and Risk of Relapse

**Figure 4:**
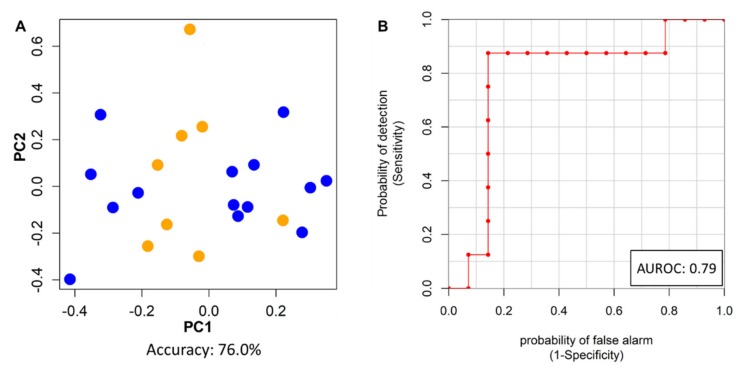
(**A**) PCA-CA of ^1^H NMR CPMG spectra of baseline plasma samples from ER(+) patients who developed disease recurrence (orange) and those who were disease free at 10 years (blue). Model LOOCV accuracy of 76.3%, *p*-value = 0.020. (**B**) ROC curve of the linear combination of IL-2 and IL-10 to distinguish relapsed and not relapsed ER(+) patients.

The immune-metabolomic profile was also explored with the aim of discriminating disease-free patients from those who developed cancer recurrence at 10 years follow-up. This analysis was performed only for ER(+) patients because within the ER(−) group too few relapses were observed (8 vs 3 respectively). Using PCA-CA-kNN on the baseline ^1^H NMR data, a good discrimination of the disease-free and relapsed patients was obtained, yielding 78.6% sensitivity, 75.0% specificity and 76.3% accuracy (Figure 4A). The permutation test for accuracy significance showed a *p*-value of 0.020. The ^1^H NMR spectral regions that mainly contribute to this discrimination were 2.23–2.21 ppm, 1.33–1.21 ppm and 0.85–0.81 ppm (Figure S6), containing signals related to lactate, threonine, acetone, HDL lipoprotein subfractions and an unknown metabolite (1.25 ppm singlet); however, none of these features reached statistical significance.

Differences in the cytokine profile showed that IL-2 and IL-10 present a near-significant association with the cancer relapse (Table S3). The linear combination of these two cytokines (96.1% IL-2 and 3.9% IL-10) well discriminated patients free from disease from those who relapsed, yielding an AUROC = 0.79, a Cliff’s delta effect size of 0.57 (large effect), and a *p*-value of 0.029 (Figure 4B).

Finally, a predictive simple “logic OR” model, based on the two IL-2 and IL-10 cytokines and the PCA-CA-kNN metabolomics model was further developed. Relapse is considered correctly predicted if either or both models can predict it. Accordingly, 100% of the relapses were correctly identified by this combined model, with only one disease free patient wrongly predicted. In summary, out of 22 ER(+) patients, one false positive (92.9% specificity) and no false negative (100% sensitivity ) were obtained, with an accuracy of 95.5%.

## 3. Discussion

The HER2-positive BC represents an aggressive phenotype with a high risk of recurrence disease and poor survival [6]. Although it accounts for only 15–20% of all BC, it constitutes a highly heterogeneous cancer disease due to its molecular and histological characteristics mainly represented by the ER expression, which is the unique feature currently used for patient stratification and for setting anti-hormonal treatments. However, the response rate to anti-EGFR target therapy in HER2-positive BC could be also influenced by the ER status, being lower in ER(+) as compared to ER(−) patients independently from the kind of target chemotherapy schedule performed [35,36,37]. So far, the reasons for this differential pattern of response and clinical outcome appear very elusive and are poorly understood.

The present study demonstrates for the first time, to the best of our knowledge, that among HER2-positive BC patients the ER status may induce specific alterations in the host biochemistry able to influence the response pattern as well as the recurrence of the disease. In this very homogeneous population of HER2-positive BC patients, baseline ^1^H NMR plasma metabolomics showed to be able to discriminate the ER(+) and ER(−) groups, since they have two distinct metabolic signatures. This result clearly indicates that differences in the tumor expression of ER significantly affect the systemic metabolism of the host, as mirrored by the differences in the metabolomic profile (Figure 1A). At the tissue level, ER(+) and ER(−) BC subtypes may have distinct metabolisms and micro-environmental modifications that depend on their differential cell growth and metabolic features [11]. These effects could be reflected on the systemic metabolomics profile as revealed in this study, suggesting that the ER status may have deep consequences also on the overall metabolism of the host. Since the population investigated resulted highly homogeneous, for both demographic and physio-pathological characteristics (Table 1), we attributed such observed circulatory metabolomics differences only to the differential cancer–host interaction addressed by the ER status. Multiparametric analysis of the NMR spectra emphasizes that the most significant metabolomics differences occur in a region of the ^1^H NMR spectra belonging to cholesterol and phospholipids signals associated to lipoprotein sub-fractions (Figure 1B and Figure S1). The reprogramming of the lipid metabolism represents one of the hallmarks of many cancers, including BC, being able to promote and sustain cancer progression [38]. Moreover, the ER status itself has been found to dramatically influence the lipid profiles, with the highest levels of membrane phospholipids detected in the most aggressive tumors [39,40]. The stimulating effect of lipids on BC tumor growth seems also be sustained by the observation that high levels of LDL-cholesterol at the time of diagnosis may have a negative prognostic value [41]. The present study pointed out that the HER2-positve/ER(+)group is characterized by high level of cholesterol-phospholipid lipoprotein subfractions that potentially could be negatively associated to the clinical outcomes of the therapeutic interventions. However, the exploratory nature of this study does not allow us to establish whether the observed circulatory phospholipid alterations derived from an aggressive tumor growth or from the host response to the tumor. 

Since the immune system is the first candidate responsible for cancer–host interaction, we investigated serum cytokines profile among the ER(−) and ER(+) groups in an attempt to reveal potential interactions between the cancer and the host immune system. Although differential cytokine patterns between the two groups are observed, these differences did not reach statistical significance, likely due to the wide inter-patient cytokine variability (Figure S2).This apparently negative result does not exclude specific immunological interactions since TILs, that characterizes the ER(−) better than the ER(+) tumor tissue [28,42], could contribute to determine local specific interactions that can be better decoded by the circulatory metabolomics profiles. 

In the present study, the pCR rate to NATC for the ER(−) and ER(+) patients was superimposable, suggesting a homogeneous pharmacological effect of the neoadjuvant treatment. However, in the ER(+) group the baseline ^1^H NMR metabolomic profile was found able to discriminate the GR patients from those who instead achieved only a partial response (Figure 2A). The lack of a similar discrimination in the ER(−) subgroup could indicate that the metabolic pathways responsible for the discrimination between GR and PR in ER(+) patients are not altered or involved (Figure 2B). The main ^1^H NMR metabolomics signatures still involved phospholipids associated to ApoB, VLDL, LDL lipoprotein sub-fractions that resulted higher in the PR patients together with ethanol, isoleucine and valine (Figure S4). In the ER(+) PR patients the higher levels of lipoprotein sub-fractions seem to support their detrimental effect on achieving a pCR. Isoleucine and valine were also found increased in the PR patients, with valine characterized by the highest diagnostic power in the discrimination between GR and PR patients (AUROC = 0.88, Figure 3A), thus suggesting an interesting association between this amino acid and the pCR response to NACT. Valine, together with leucine and isoleucine, belong to the branched-chain amino acids class known to play an important role in the alternative energy supply of cancer cells [43,44]. It can be hypothesized that the lower level of valine in the plasma of the GR patients may contribute to limit the cancer cell viability, improving the pharmacological effect of NATC. Interestingly, GR patients showed a specific cytokine profile characterized by high levels of TNF-α, able to discriminate, like valine, GR from PR patients in ER(+) group (AUROC = 0.84 Figure 3B). High levels of this cytokine were frequently reported in the serum of BC patients as compared to healthy women where TNF-α is generally lower or not detected [45]. TNF-α is a pro inflammatory cytokine that may have different effects on BC cells. Although TNF-α can be found at BC tissue level, to date it is unknown whether the TNF-α surrounding the tumor is produced and released by cancer cells as an evasion strategy from the host immune response or whether it is secreted by cells of the immune system that infiltrate the tumor tissue. Recent evidences reported that HER2-overexpressing BC cells are susceptible to apoptosis induced in vitro by T helper 1 cytokines as TNF-α [46,47]. Through a complex regulatory network, after its receptor activation, TNF-α has been found to induce apoptosis or necrosis but also an opposite effect inducing cell growth, invasion or propagation of cancer cells [48,49]. Conversely, antigen-specific CD8+ T cells use TNF-α as part of their anti-tumor effector arsenal to better support the antibody-dependent cell-mediated cytotoxicity (ADCC) driven by trastuzumab. The higher level of circulatory TNF-α observed in the GR patients within the ER(+) group seems to support the hypothesis of a more proficient immune system in these patients. 

The most relevant metabolomic and immunological markers, represented by valine and TNF-α, embody independent information, and when they are combined the discrimination power of the model increases significantly (AUROC = 0.92, Figure 3C). This result highlights that the integration of baseline immune-metabolomics information may play a key role for the prediction of the response to NATC, contributing to better identify the HER2-positive/ER(+) patients that achieve the best pharmacological benefit. 

Besides the response to NATC treatment, the rate of relapse for the HER2-positive patients enrolled in this study resulted more favorable for the ER(−) than ER(+) patients, being 14% and 36% respectively. These findings were in agreement with previous reports that indicate an increased rate of relapse for the ER(+) patients in spite of the use of adjuvant therapy with tamoxifen and/or aromatase inhibitors [35]. In this study it was possible to identify metabolomic and immunological signatures associated with the increased risk of disease recurrence for the HER2-positive/ER(+) patients, contributing to make the immune-metabolomic profile a potential independent risk factor of relapse. Thus, the baseline metabolomics profile, before NATC treatment and surgery, contains information useful to identify the patients who would relapse within a 10 years follow-up with satisfactory accuracy (Figure 4A). Analogously, the cytokine profile provides interesting information since lower IL-2 and IL-10 baseline levels were found able to identify the relapsed patients with high sensitivity and specificity (Figure 4B). These results are corroborated by previous observations indicating that particularly low levels of IL-2 during follow-up were associated with higher risk of relapse [50], while other investigations noticed that IL-10 seems to decrease during neoadjuvant chemotherapy in PR patients [51]. Thus, the low levels of IL-2 and IL-10 in ER(+) BC relapsed patients could reflect a reduced ability to exert a proper response to therapy. Indeed, both cytokines contribute to the modulation of the patients’ immune system, involved in the response to trastuzumab, taxanes as well as hormonal therapy. The higher levels of IL-2 observed in disease-free patients can contribute to make the effect of NATC more stable by promoting the recruitment and activation of natural killer (NK) and cytotoxic CD8+ T lymphocytes at the tumor site, improving the ADCC mediated by trastuzumab [52,53]. If the IL-2 effect consists in the activation of the host immune system, the role of IL-10 is not so clear, as IL-10 is a pleiotropic cytokine that at low concentrations exhibits tumor-promoting activity, while at high levels it shows an anti-tumor effect [54,55]. Both IL-2 and IL-10 were found to increase the cytotoxic activity of anti-tumor CD8+ T cells in vitro [56], suggesting a potential synergistic effect for the two cytokines. In the present study the linear combination model of IL-2 and IL-10 was able to improve the discrimination (AUROC = 0.79) of the relapsed patients from those who were disease-free, supporting that a properly activated immune system, as revealed by the higher level of IL-2 and IL-10, may contribute to decrease the risk of disease relapse. The discrimination power of the IL-2 and IL-10 model significantly improved with the integration of individual metabolomics information, allowing the correct identification of all the relapsed patients (100% sensitivity) with only one free-disease patient wrongly classified (92.9% specificity). This result indicates that immune and metabolomics profiles may represent independent markers to predict disease relapse, and that their combination can find application for the identification of patients with high-risk of recurrence with the aim of ensuring them an extended adjuvant targeted therapy.

The low sample size of this exploratory study may lead to a lack of sufficient statistical power for multivariate analysis or stratification analysis, thus further studies with larger sample sizes are needed to overcome these caveats and validate the findings. Conversely, the current study has the important strength to be a mono-institutional investigation based on a very homogeneous population of HER2-positive BC patients all treated with the same NATC schedule. Although the results should be evaluated in light of these limitations and strengths, this translational study seems to indicate that ER(+) and ER(−) HER2-positive BC should be considered as two distinctive diseases able to induce different host interactions, as mirrored by the circulatory ^1^H NMR metabolomic profiles.

## 4. Materials and Methods

### 4.1. Patients’ Population and Therapy

The patients enrolled in this study belong to a single arm, phase II mono-institutional study. Forty-three patients were consecutively enrolled in this investigation from July 2006 to October 2010. All patients had histologically confirmed locally advanced HER-2 positive BC (UICC stage II–III) (Table 1) and underwent NATC with trastuzumab (loading dose 4 mg/kg intravenously, then 2 mg/kg) in combination with weekly paclitaxel (80 mg/m^2^ on day 1, 8, 15, 22) every 21 days. In the presence of clinical response, 3 further cycles were administered to obtain pCR. After NATC completion, patients underwent primary surgery (mastectomy or conservative treatment) and axillary node dissection when indicated. After surgery, adjuvant trastuzumab-paclitaxel was administered for 3 cycles and trastuzumab alone every 3 weeks for 1 year. Radiation and/or hormonal therapy was performed if indicated. This study (NCT02307227) was conducted according to the ethical principles of the Declaration of Helsinki and approved by the local Ethical Committee. The study was approved by the Institutional Ethical Committee on 24 May 2006, Ethic code cro-18-2006. The study was also registered in clinicaltrial.gov with 02307227 NCT identification number for more information as described in the manuscript. Written informed consent was obtained from all patients.

### 4.2. Immunohistochemistry: Hormonal Receptor Status Score

ER together with PgR immunohistochemical analysis was performed on 4 mm section of each paraffin block from biopsy or tumor tissues adequately stained with haematoxylin-eosin. The so prepared tissue samples were deactivated from endogenous peroxidases by incubating with streptavidin-horseradish peroxidase (Dako) 1:100 hydrogen peroxide/diaminobenzidine chromogen. Staining with primary monoclonal antibodies against ER and PgR (6 F11 MAb and 1E2 from Ventana Medical Systems, Inc., Tucson, AZ MAb respectively from DAKO) was done overnight at room temperature followed by a 30 min incubation with biotinylated rabbit anti-mouse antibody (Dako). Osmium tetroxide 0.2% incubation for 30 s and methyl green counter stain were used as a signal enhancer. Finally, samples were float-mounted on adhesive-coated glass slides. Normal human endocervix and stomach tissues were used as positive controls, while negative controls consisted of substituting non-immune mouse antibodies [57]. Controls were included with each batch of slides for immunohistochemistry (IHC) analysis. The immuno-stained slide samples were entirely evaluated by light microscopy and scored on the basis of the estimated proportion of nuclear positively staining tumor cells according to Allred et al., 1998 (0, none; 1,1/100; 2, 1/100 to 1/10; 3, 1/10 to 1/3; 4, 1/3 to 2/3; 5, >2/3). The proportion score and the intensity score are classified in six and four grades, respectively 0–5 and 0–3, then the total score is assessed in eight grades (0 and 2–8) [58]. Tumors with IHC total score of 3 were reported as positive. A score index of 0, 1, 2, and 3 was used, corresponding to negative, weak, moderate, and strong staining intensity respectively, and the percentage of positive cells at each intensity was estimated subjectively. 

### 4.3. Sample Collection

Plasma-ACD and serum were acquired from whole blood samples collected under fasting conditions from each patient enrolled before the NACT. They were obtained after blood centrifugation at 2100 rpm and stored at −80 °C until analysis.

### 4.4. Serum Cytokine profile Analysis

The immune cytokines panel used in the current investigation was designed to mainly reveal T-cell activity. The cytokines selection was based on the following factors: (1) detectability in serum samples and (2) activators of anti-tumor T lymphocytes, (3) mediators of the inflammatory response or (4) immunosuppressive factors. Levels of interleukin (IL)-1α, IL-1β, IL-2, IL-6, IL-8, IL-10, IL-12p70, TNF-α, and granulocyte macrophage colony-stimulating factor (GM-CSF) were evaluated using the SearchLight^®^ multiplex arrays (Food and Drug Administration approved, Aushon Biosystems, TEMA Ricerca, Bologna, Italy) according to the manufacturer’s instructions. Briefly, custom human 8-plexarray and human 1-plexarray (for GM-CSF detection) with pre-spotted cytokine-specific antibodies were used. Standards or pre-diluted samples were added in duplicate and, after 1 h of incubation at room temperature and 3 washes, the biotinylated antibody reagent was added to each well. After 30 min incubation at room temperature and 3 washes, block solution was added to stabilize the signal. The addition of Streptavidin-HRP Reagent and SuperSignal^®^ Substrate (TEMA Ricerca, Bologna, Italy), and the acquisition of luminescent signal with a cooled Charge Coupled Device camera, together with data analysis and processing, were performed by TEMA Ricerca laboratories’ customer service (Bologna, Italy). TGF-β1 serum levels were assessed through ELISA (DRG Instruments GmbH, Marburg, Germany) according to manufacturer’s instructions. Pre-diluted samples and standards underwent appropriate acidification and neutralization before testing. Briefly, pre-treated standards, controls and samples were dispensed into wells in duplicates and plates were incubated overnight at 4 °C. After 3 washes, antiserum was added to the wells and incubated for 120 min at room temperature, the plate was rinsed 3 times and anti-mouse biotin (enzyme conjugate) was dispensed and incubated for 45 min. After 3 washes, the enzyme complex was added to the wells, the plates were incubated 45 min and washed 3 times. After the addition of substrate solution for 15 min, the reaction was stopped and the absorbance at 450 ± 10 nm was determined with a microtiter plate reader (Bio-Tek Instruments, Winooski, VT, USA).

### 4.5. ^1^H NMR Metabolomic Analyses

All plasma-ACD samples were analyzed following the standard operating procedures developed by our laboratory [59]. Frozen plasma samples were thawed at room temperature and shaken before use; then 350 µL of a sodium phosphate buffer (70 mM Na_2_HPO_4_·7H_2_O; 6.1 mM NaN_3_; 4.6 mM sodium trimethylsilyl [2,2,3,3-^2^H_4_]propionate (TMSP), 20% (v/v) ^2^H_2_O in H_2_O; pH 7.4) was added to 350 µL of each sample, and the mixture was homogenized by vortexing for 30 s. A total of 600 µL of this mixture was transferred into a 5 mm NMR tube for the NMR analysis.

The ^1^H NMR spectra were acquired using a Bruker 600 MHz NMR spectrometer operating at 600.13 MHz proton Larmor frequency and equipped with a 5mm PATXI ^1^H-^13^C-^15^N and ^2^H-decoupling probe including a z axis gradient coil, an automatic tuning-matching (ATM) functionality and an automatic refrigerated sample changer (SampleJet^®^, Bruker BioSpin, Rheinstetten GmbH, Germany). A BTO 2000 thermocouple served for temperature stabilization (approximately 0.1 K at the sample level). Before each measurement, temperature equilibration at 310 K was obtained by keeping the sample at least 5 min inside the NMR probehead.

For each plasma sample two one-dimensional ^1^H NMR spectra were acquired: a standard spin echo Carr-Purcell-Meiboom-Gill [60] (CPMG) pulse sequence (32 scans, 73,728 data points, spectral width of 12,019 Hz, total spin echo of 80 ms, acquisition time of 3.1 s, and relaxation delay of 4 s) was used to selectively detect low molecular weight metabolites. A standard nuclear Overhauser effect spectroscopy pulse sequence NOESY 1Dpresat (32 scans, 98,304 data points, spectral width of 18,028 Hz, acquisition time of 2.7 s, relaxation delay of 4 s and mixing time of 0.01 s) was instead applied to obtain spectra in which signals from both low and high molecular weight molecules are present. The CPMG spectra were used for the multivariate analysis, since in NOESY spectra it was difficult to remove the signals of the two additives, dextrose and citrate. Conversely, NOESY spectra were used for metabolite quantification (see below in Statistical analysis paragraph).

Free induction decays were multiplied by an exponential function equivalent to a 0.3 Hz line-broadening factor before applying Fourier transform. Transformed spectra were automatically corrected for phase and baseline distortions and calibrated (anomeric glucose signal 5.24 ppm) using TopSpin 3.2 (Bruker BioSpin GmbH, Rheinstetten, Germany). 

Each 1D CPMG spectrum in the range 0.2–10.00 ppm was segmented into 0.02 ppm chemical shift bins, and the corresponding spectral areas were integrated using the AssureNMR software (Bruker BioSpin GmbH, Rheinstetten, Germany). To remove the interference effect due to the additives present in the samples [61], the region between 6.00 and 4.39 ppm containing the residual water signal and the regions containing signals of dextrose and citrate (3.91–3.71 ppm, 3.55–3.23 ppm, 2.69–2.51 ppm, and 1.19–1.15 ppm) were removed, and the dimension of the system was reduced to 368 bins. Total integral normalization was applied on the remaining bins prior to perform statistical analysis. 

### 4.6. Statistical Analysis

Data analyses were performed in the “R” open source software environment [62]. Multivariate analysis was conducted on binned data. Data reduction was carried out by projection into a principal component analysis (PCA) subspace. Only the minimum number of components that maximized the accuracy was retained in the model and the canonical analysis (CA) was applied to obtain the supervised separation of the groups of interest [63]. For the purpose of classification, K-nearest neighbors (k-NN) method applied on the PCA-CA scores was used. For each model, accuracy, sensitivity and specificity, calculated according to the standard definitions, were assessed by means of a Leave-One-Out cross-validation scheme (LOOCV, R script developed in-house). Briefly, the algorithm is trained on all the data except for one sample, and a prediction is made for that sample. The procedure is repeated for each sample, and sensitivity, specificity and accuracy for the classification is assessed. The significance of the classification results was assessed by means of a permutation test, using 10^3^ permutations.

Metabolite and lipoprotein concentrations were estimated on ^1^H NOESY spectra using the Bruker IVDr platform [64]. Receiver operating characteristic (ROC) analyses, calculated using the R package “caTools”, were applied to assess whether metabolite and/or cytokine concentrations could differentiate the groups of interest. The Wilcoxon-Mann-Whitney test was chosen to infer differences between the groups, and a *p*-value adjusted with the Benjamini and Hochberg correction-lower than 0.05 was deemed significant. Furthermore, for each analyzed variable (metabolites and cytokines), effect size using Cliff’s delta was calculated by means of the R package “effsize”.

For demographic and clinical characteristics, t-test was used for comparison between numeric variables, and the chi-square test for comparison between categorical variables.

## 5. Conclusions

The results of this exploratory translational study highlight the importance of integrating the circulatory immune-metabolomics information with current conventional stratification criteria in HER2-positive BC patients. ER status in HER2-positive BC patients was found to induce significant changes in the host circulatory metabolome with important implications for the pCR to NACT and for the overall clinical outcome.

## Figures and Tables

**Table 1:** Demographic and clinical characteristics of the HER2-positive patients as a function of the ER status.

Characteristic	ER (−) *n* = 21	ER (+) *n* = 22	*p*-Value
**Age (years)**			
Median (range)	48 (28–70)	49 (23–68)	0.878 *
**BMI (Kg/m^2^)**			
Mean ± SD	24.1 ± 9.0	26.6 ± 6.1	0.19
**Stage**			
IIA	4	2	
IIB	13	14	0.443 ^#^
IIIA	4	4	
IIIB	0	2	
**Grade**			
G2	4	4	
G3	16	17	0.444
GX ^§^	1	1	
**Ki67**			
<20	10	9	0.097
≥20	11	13	
**Pathological response**			
Complete	13	11	0.432
Partial **Disease Recurrence**	8	11	
Yes	3	8	0.097

^§^ unclassified, * *t*-Test, ^#^ chi-squared test.

## Supplementary Materials

The following are available online: https://www.mdpi.com/2072-6694/12/2/314/s1. Figure S1: Comparison between ER(−) and ER(+) BC patients on baseline plasma samples via 1H NMR metabolomics; Figure S2: Concentration of 10 cytokines in the serum HER2-positive ER(+) and ER(−) BC patients. Mean ± Standard Deviation (SD). n; number of patients for which each cytokine was quantify reported parenthesis. NS; No statistical significative. Figure S3: Analysis of baseline plasma samples via 1H NMR metabolomics to compare GR and PR to NATC in ER(+) (A) and ER(−) (B) patients; Figure S4: Comparison between GR and PR subgroups within HER2-positive/ER(+) patients at baseline via 1H NMR metabolomics; Figure S5: ROC curves of valine (A) and TNF-α (B) diagnostic power to distinguish ER(−) patients who achieve pathological response (GR) from those who achieve only a partial response (PR) to NATC treatment. The ROC curve of the combined linear model VAL+TNFα (C) shows improved (not relevant) diagnostic power; Figure S6: Baseline 1H NMR metabolomic profile comparison between patients who developed disease recurrence and those who were found disease free at 10 years; Table S1: Analysis of cytokines in ER(+) patients to discriminate the patients who achieve pathological response (GR) from those who achieve only a partial response (PR) to NATC treatment; Table S2: Analysis of cytokines in ER(−) patients to discriminate the patients who achieve pathological response (GR) from those who achieve only a partial response (PR) to NATC treatment; Table S3: Analysis of cytokines in ER(+) patients to discriminate disease free patients from those who relapsed.
