# Supplementary material for: Effect of Estrogen Receptor Status on Circulatory Immune and Metabolomics Profiles of HER2-Positive Breast Cancer Patients Enrolled for Neoadjuvant Targeted Chemotherapy
Source: Cancers (Basel). 2020 Jan 29;12(2):314. doi: 10.3390/cancers12020314 (PMC7072610; doi:10.3390/cancers12020314)
Supplement: Supplementary file 1 [file cancers-12-00314-s001.pdf]

Supplementary Materials

Contents

Figure S1. Comparison between ER(−) and ER(+) BC patients on baseline plasma samples via ^{1}H NMR metabolomics.

Figure S2. Concentration of 10 cytokines in the serum HER2-positive ER(+) and ER(−) BC patients. Mean ± Standard Deviation (SD). n; number of patients for which each cytokine was quantify reported parenthesis. NS; No statistical significative.

Figure S3. Analysis of baseline plasma samples via ^{1}H NMR metabolomics to compare GR and PR to NATC in ER(+) (A) and ER(−) (B) patients.

Figure S4. Comparison between GR and PR subgroups within HER2-positive/ER(+) patients at baseline via ^{1}H NMR metabolomics.

Figure S5. ROC curves of valine (A) and TNF-α (B) diagnostic power to distinguish ER(−) patients who achieve pathological response (GR) from those who achieve only a partial response (PR) to NATC treatment. The ROC curve of the combined linear model VAL+TNF-α (C) shows improved (not relevant) diagnostic power.

Figure S6. Baseline ^{1}H NMR metabolomic profile comparison between patients who developed disease recurrence and those who were found disease free at 10 years.

Table S1. Analysis of cytokines in ER(+) patients to discriminate the patients who achieve pathological response (GR) from those who achieve only a partial response (PR) to NATC treatment.

Table S2. Analysis of cytokines in ER(−) patients to discriminate the patients who achieve pathological response (GR) from those who achieve only a partial response (PR) to NATC treatment.

Table S3. Analysis of cytokines in ER(+) patients to discriminate disease free patients from those who relapsed.

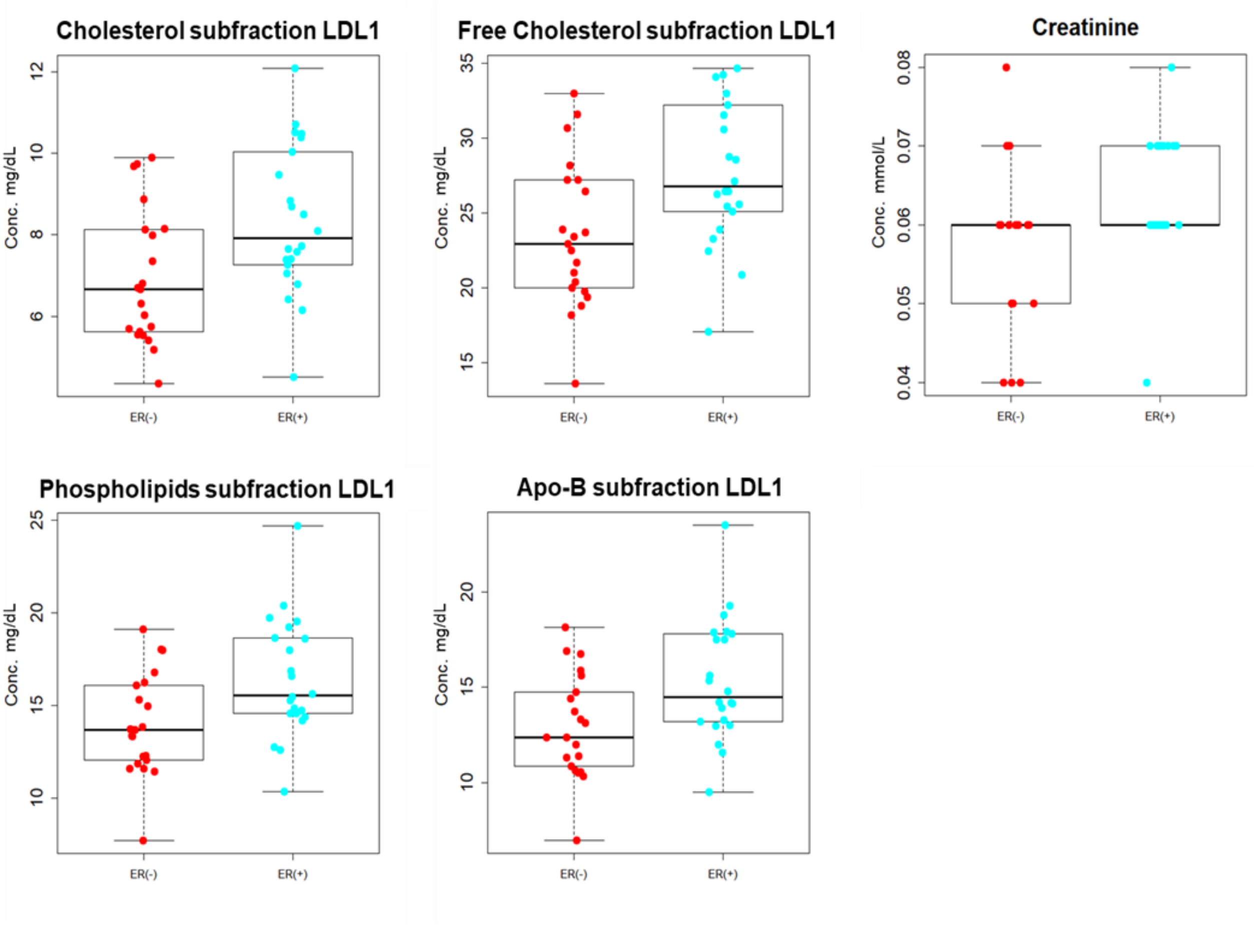

Figure S1. Comparison between ER(−) and ER(+) BC patients on baseline plasma samples via ^{1}H NMR metabolomics. Metabolites and lipid fractions that significantly contribute to the discrimination are reported.

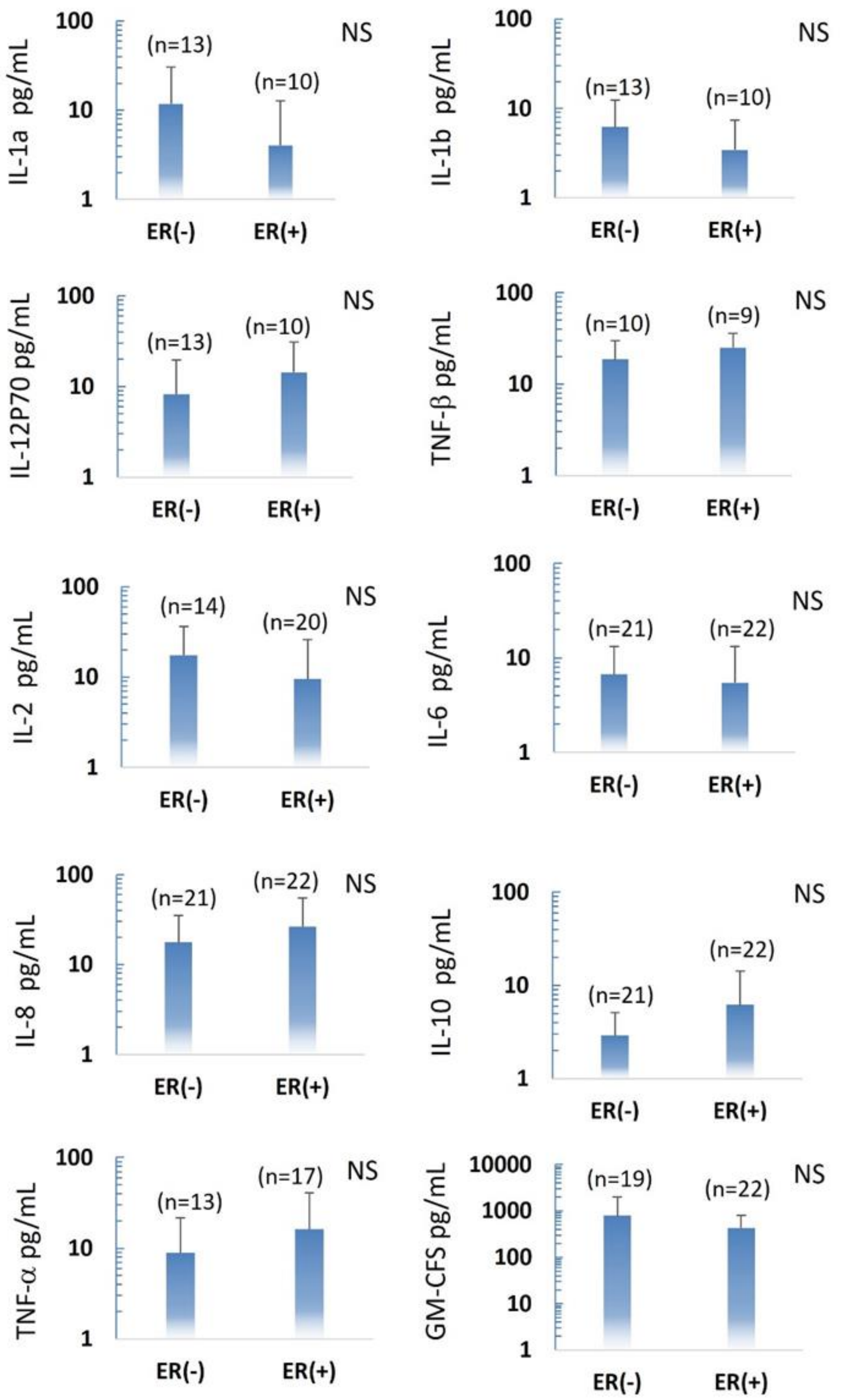

Figure S2. Concentration of 10 cytokines in the serum HER2-positive ER(+) and ER(-) BC patients. Mean + Standard Deviation (SD). n: number of patients for which each cytokine was quantify reported parenthesis. NS: No statistical significative.

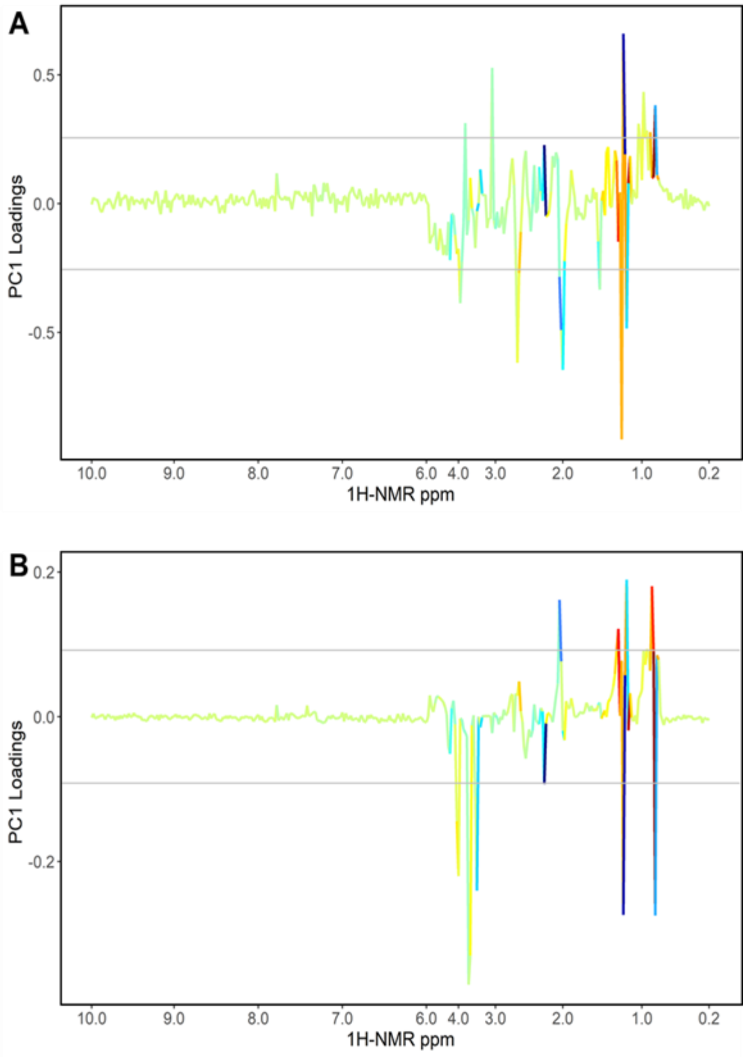

Figure S3. Analysis of baseline plasma samples via ^{1}H NMR metabolomics to compare GR and PR patients to NATC in ER(+) (A) and ER(-) (B) patients; Loading plots of the first component of the PCA-CA calculated on the CPMG spectra.

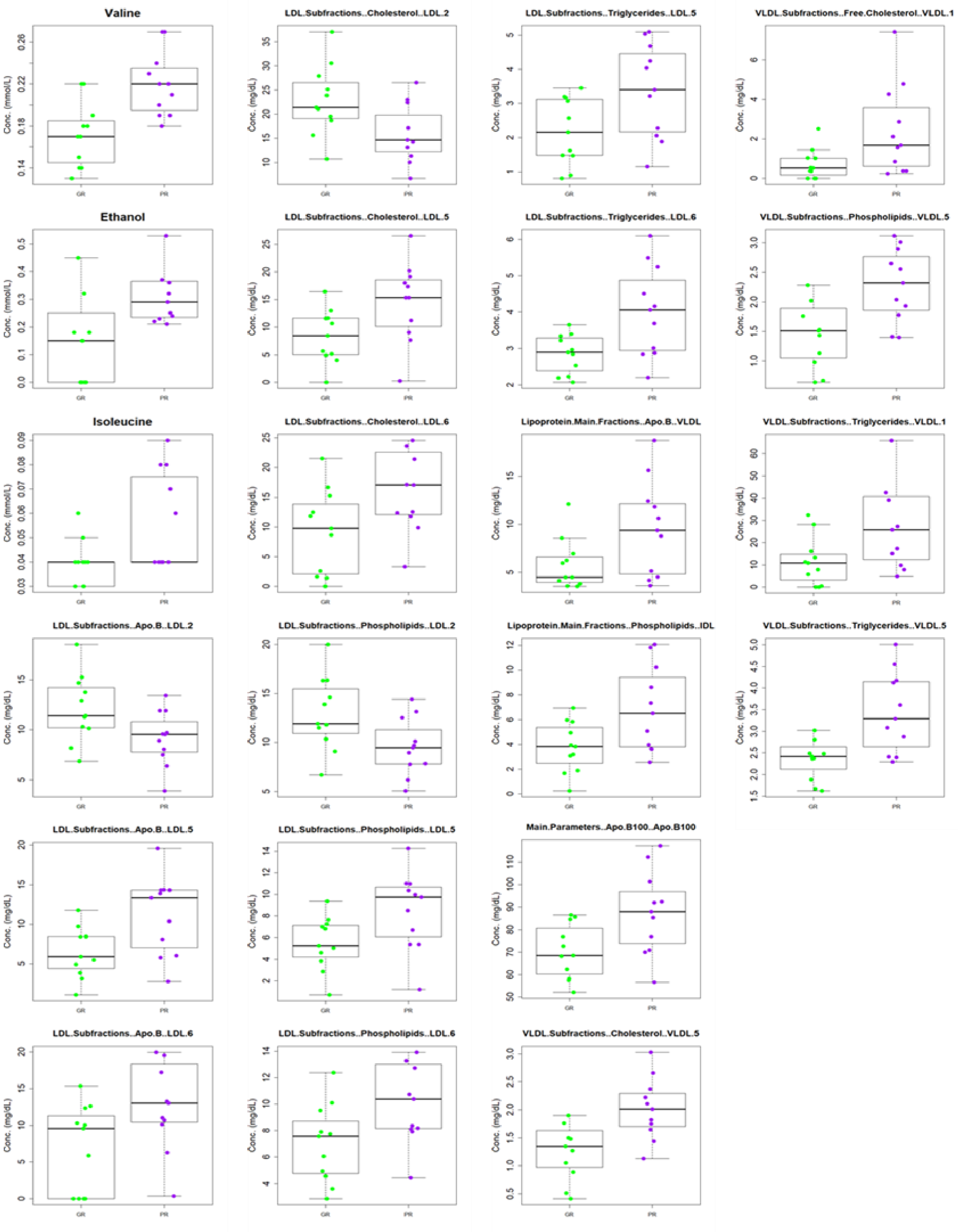

Figure S4. Comparison between GR and PR ER(+) patients at baseline via ^{1}H NMR metabolomics. Metabolites and lipid fractions that significantly contribute to the discrimination are reported.

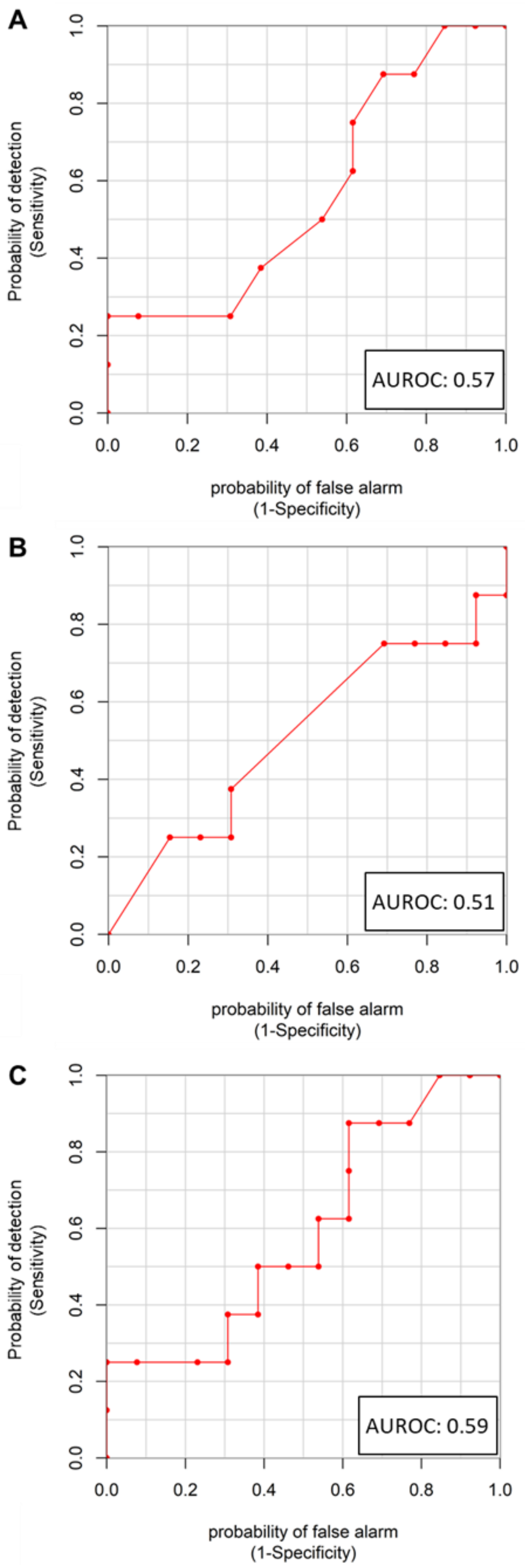

Figure S5. ROC curves of valine (A) and TNF-α (B) diagnostic power to distinguish ER(-) patients who achieve pathological response (GR) from those who achieve only a partial response (PR) to NATC treatment. The ROC curve of the combined linear model VAL+TNF-α (C) shows improved (not relevant) diagnostic power.

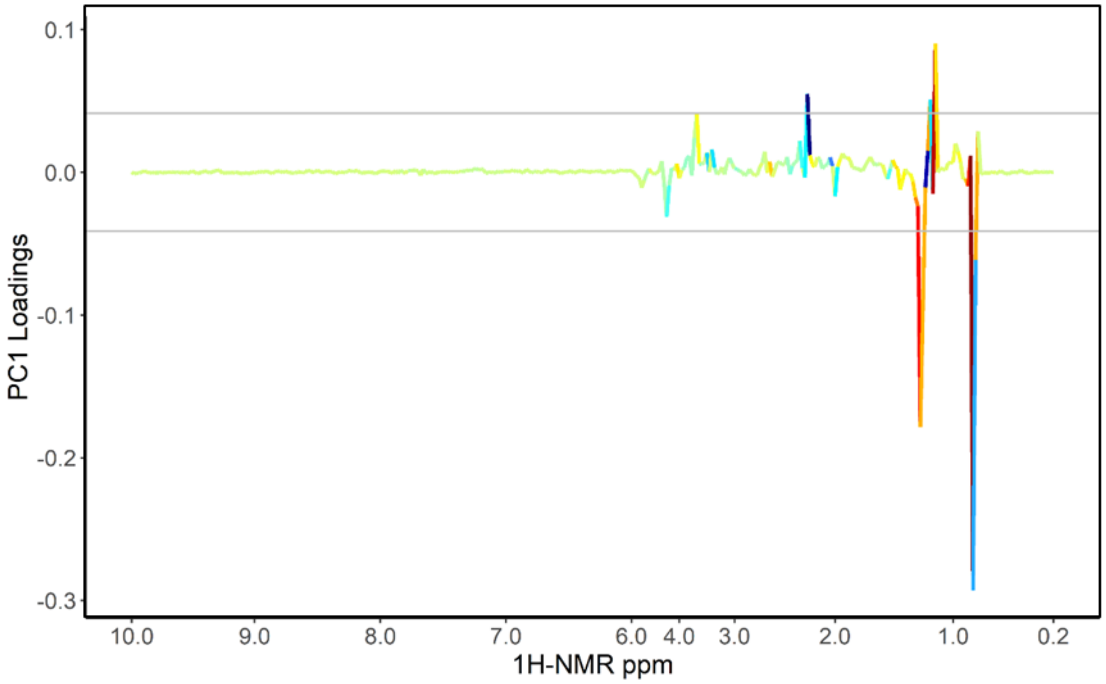

Figure S6. Baseline ^{1}H NMR metabolomic profile comparison between ER(+) patients who developed disease recurrence and those who were disease free at 10 years. Loading plots of the first component of the PCA-CA calculated on the CPMG spectra.

Table S1. Analysis of cytokines in ER(+) patients to discriminate the patients who achieve pathological response (GR) from those who achieve only a partial response (PR) to NATC treatment.

	AUROC	P-value	P-value adjusted with FDR	Cliff's delta effect size
**IL-2**	0.70	0.12	0.37	0.40
**IL-6**	0.52	0.90	0.90	0.04
**IL-8**	0.64	0.29	0.50	-0.27
**IL-10**	0.53	0.85	0.90	-0.06
**TNF-α**	0.84	0.01	0.04	0.68
**GM-CSF**	0.62	0.33	0.50	0.23

Table S2. Analysis of cytokines in ER(−) patients to discriminate the patients who achieve pathological response (GR) from those who achieve only a partial response (PR) to NATC treatment.

	AUROC	P-value	P-value adjusted with FDR	Cliff's delta effect size
**IL-2**	0.58	0.58	0.70	-0.15
**IL-6**	0.61	0.46	0.68	-0.21
**IL-8**	0.79	0.03	0.15	0.58
**IL-10**	0.74	0.08	0.15	0.48
**TNF-α**	0.51	0.94	0.94	0.03
**GM-CSF**	0.73	0.07	0.15	-0.46

Table S3. Analysis of cytokines in ER(+) patients to discriminate the patients who free from disease and relapsed patients.

	AUROC	P-value	P-value adjusted with FDR	Cliff's deltaeffect size
**IL-2**	0.77	0.04	0.12	-0.54
**IL-6**	0.63	0.32	0.48	-0.27
**IL-8**	0.50	1.00	1.00	-0.01
**IL-10**	0.79	0.03	0.12	-0.57
**TNF-α**	0.56	0.65	0.79	-0.13
**GM-CSF**	0.69	0.12	0.23	0.38
